# Comparative Study on Structural and Transport Properties of SSC and LSC PFSA Ionomers in PEMFCs with Coexistence of O_2_ and N_2_: Molecular Dynamics Simulation Approach

**DOI:** 10.3390/membranes15110324

**Published:** 2025-10-22

**Authors:** Guanghua Wei, Jingjing Huang, Lina Yu, Jinghao Zhou, Jiabin You, Zhu Ling, Shenrong Ye, Junliang Zhang

**Affiliations:** 1 SJTU Paris Elite Institute of Technology, Shanghai Jiao Tong University, Shanghai 200240, China; ghwei@sjtu.edu.cn (G.W.); lingzhu@sjtu.edu.cn (Z.L.); olivier.9928@sjtu.edu.cn (S.Y.); 2 Shanghai Zhuoxing Technology Co., Ltd., Shanghai 200241, China; jane0730@alumni.sjtu.edu.cn; 3 Commercial Vehicle Development Institute, FAW Jiefang Automotive Co., Ltd., Changchun 130011, China; yulina@rdc.faw.com.cn; 4 Institute of Fuel Cells, Shanghai Jiao Tong University, Shanghai 200240, China; zhoujinghao922@sjtu.edu.cn (J.Z.); jiabinyou@163.com (J.Y.); 5 MOE Key Laboratory of Power & Machinery Engineering, Shanghai Jiao Tong University, Shanghai 200240, China

**Keywords:** ionomer, short-side-chain perfluorosulfonic acid, catalyst layer, proton exchange membrane fuel cell, molecular dynamics simulations

## Abstract

Efficient O_2_ transport through the ionomer film in cathode catalyst layers (CCLs) is a critical factor for the output performance of proton exchange membrane fuel cells (PEMFCs), yet the molecular mechanisms of gas transport in ionomers remain elusive. Herein, molecular dynamics (MDs) simulations are employed to investigate short-side-chain (SSC) and long-side-chain (LSC) perfluorosulfonic acid (PFSA) ionomers on Pt/C surfaces with the coexistence of O_2_/N_2_. The results reveal that the side-chain structures significantly modulate the ionomer nanostructures and gas transport. SSC ionomers form compact hydrophobic domains and more interconnected hydrophilic–hydrophobic interfaces, thereby facilitating more efficient O_2_ transport pathways than LSC ionomers, particularly at low hydration (λ = 3). At high hydration (λ = 11), swelling of water domains attenuates these structural disparities and becomes the dominant factor governing gas transport. In addition, O_2_ diffusion consistently exceeds that of N_2_, while the diffusion coefficients of O_2_, N_2_ and H_3_O^+^ become larger at high hydration. Collectively, these findings demonstrate the structural advantages of SSC ionomers in facilitating coupled oxygen and proton transport, offering molecular-level insights to inform the rational design of high-performance PEMFCs.

## 1. Introduction

Proton exchange membrane fuel cells (PEMFCs) are widely regarded as promising energy conversion technologies due to their high efficiency, low operating temperature, environmental compatibility, and rapid start-up capability [1,2,3]. Nevertheless, the high cost associated with platinum-group-metal (PGM) catalysts, which are required to overcome the sluggish kinetics of the oxygen reduction reaction (ORR) at the cathode, remains a critical barrier to large-scale commercialization of PEMFCs. Consequently, reducing catalyst loading while sustaining high performance has become a central objective in related research and development [4,5]. It is well-acknowledged that lowering Pt loading causes drastic increase in local oxygen transport resistance within the cathode catalyst layers (CCLs), primarily due to concentration loss at high-current density [6,7]. In particular, the nanostructure of ionomer films at the triple-phase boundaries (TPBs) plays a crucial role in regulating the gas transport, water distribution, and proton conduction, yet a comprehensive understanding relating interfacial structure and performance remains a challenge [8].

While alternatives emerge [9], perfluorosulfonic acid (PFSA) ionomers still serve as the typical proton conductors and binders in CCLs, owing to their excellent proton conductivity, mechanical strength, and chemical stability [10]. However, under low Pt loading conditions, oxygen transport resistance within the CCLs becomes increasingly critical, as ionomer films covering the catalyst nanoparticles must simultaneously ensure efficient proton conduction and minimizing blockage of reactant gas from reaching the active sites [11,12]. To meet the U.S. DOE Multi-Year Program Plan’s demand for developing ionomers with high oxygen permeability [13], the chemical structure and morphology of ionomers are considered as key factors in regulating mass transport efficiency [14,15,16,17].

Tailoring the side-chain structures of PFSA ionomers has been regarded as a promising strategy to alleviate oxygen transport resistance while elevating the proton conduction. Studies show growing attention to the application of short-side-chain (SSC) PFSA [18,19], such as Aquivion. Compared to conventional long-side-chain (LSC) PFSA exemplified by Nafion, SSC ionomers feature higher sulfonic acid group density and shorter side-chains. Extensive experimental studies have reported SSC ionomers’ improved water uptake [20,21,22], distinct morphology [23,24,25], enhanced proton transport under low humidity [25,26,27,28], and other properties including durability [29] and mechanical properties [30]. In addition, SSC ionomers have demonstrated considerable potential in optimizing mass transport. For example, Garsany et al. reported that SSC-based catalyst layers possess over three-times higher micropore volume, contributing to reduced mass transport losses [31]. Park et al. observed via STEM thinner and more homogeneous ionomer films on Pt/C, which lower resistances of both oxygen and proton transport [32]. Similarly, Li et al. found that SSC PFSA enables porous structures with uniform coverage on the catalysts, supported by limiting current experiments and molecular dynamics (MDs) simulations [33]. Kwon et al. further showed through MD simulations that denser sulfonic acid group distribution in Aquivion promotes well-developed water channels and enhances simultaneous diffusion coefficients of oxygen, water, and hydronium ions [34,35].

It is worth noting that the gas composition can significantly influence the gas transport process through the ionomer film. Shen et al. reported an increase of 44.7% in local resistance as the oxygen mole fraction increased from 1% to 8% in the O_2_/N_2_ mixture, highlighting the sensitivity of transport to gas composition [36]. Ban et al. conducted MD simulations of H_2_/O_2_/N_2_ mixture with molar fraction equal to 5:1:4, and found that N_2_ molecules can suppress O_2_ adsorption within Nafion ionomers [37]. You et al. further investigated O_2_/N_2_ permeation through hydrated ionomer films with varying hydration levels, revealing higher diffusion coefficients and solubility for O_2_ compared to N_2_ [38]. Furthermore, analysis of bond lengths suggested that O_2_ preferentially diffuses through interfacial or hydrophobic regions of hydrated ionomers. Despite these insights, studies on how side-chain structures modulate gas transport in the coexistence of O_2_/N_2_ remain limited. To address this gap, we conducted a comparative study on hydrated SSC and LSC PFSA ionomers in the CCL under the mixed O_2_/N_2_ conditions. The nanostructures of ionomer films near TPBs are first analyzed. Then, gas distribution and water morphology are investigated to understand the transport process. Finally, the diffusion coefficients of O_2_, N_2_ and H_3_O^+^ in CCLs of different side-chains and hydration conditions are discussed.

## 2. Methods

### 2.1. Simulation Models

**Figure 1:**
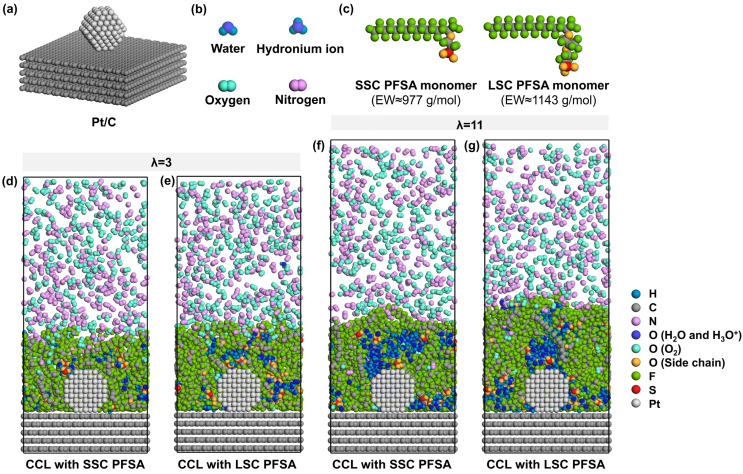
Schematic illustration of model components, (**a**) Pt/C, (**b**) water, hydronium ion, oxygen and nitrogen, (**c**) monomer of SSC and LSC PFSA chains with EW ≈ 977 and 1143 g/mol, (**d**,**e**) cross-sectional view of CCLs with SSC and LSC PFSA under λ = 3, (**f**,**g**) cross-sectional view of CCLs with SSC and LSC PFSA under λ = 11 (the wall at the top is hidden for simplicity).

To simulate the ionomer nanostructure and the local O_2_/N_2_ transport near TPBs, the model consists of carbon support, Pt particles, PFSA chains with different side-chains, water molecules, hydronium ions, and oxygen molecules. The carbon support is composed of five layers of ideal graphene sheets with an in-plane size of 51.13 Å × 49.20 Å and a total thickness of 13.60 Å. As shown in Figure 1a, a ~2 nm Pt particle [39,40] with a cuboctahedral morphology is attached to the center of the surface of carbon support to model the catalyst containing six (100) planes and eight (111) planes [41] near a stable TPB. The Pt/C layer comprising 5025 carbon atoms and 201 Pt atoms in total is fixed at the bottom of the box.

Then, the ionomer thin film which covers the Pt/C surface is modeled by 8 PFSA chains and corresponding H_2_O molecules and H_3_O^+^ ions. As presented in Figure 1c, SSC and LSC PFSA chains were modeled in this study, representing PFSA chains of EW ≈ 977 and 1143 g/mol [42,43]. Each PFSA chain was set to a polymerization degree of 10 with identical hydrophobic backbone matrix. Sulfonic groups are assumed to be fully deprotonated [44]; thus, 80 H_3_O^+^ were added to the system to maintain the charge neutrality. Two hydration conditions (the ratio of the number of H_2_O molecules and H_3_O^+^ ions to that of sulfonic groups) of λ = 3 and 11 were chosen to simulate different operating conditions of low- and high-relative humidities (~40% and ~90% [45]) at 353 K, representative for PEMFCs’ component design targets [25,46]. Therefore, 160 and 800 H_2_O molecules were added to the system, respectively.

### 2.2. MD Parameters and Procedures

All-atom MD simulations were carried out in this study. The COMPASS force fields [47,48] were adopted due to their accurate prediction and reliability in relevant molecular studies of PEMFCs [49,50]. All the MD simulations were employed with a time step of 1.0 fs. The Nosé–Hoover thermostat [51] with a Q ratio of 0.1 was utilized for controlling the temperature. The particle–particle–particle mesh (PPPM) summation method [52] was implemented to calculate the long-range interactions with an accuracy of 1.0 × 10^−3^ kcal/mol, while the cutoff distance of van der Waals (vdW) interactions was set at 12.5 Å. Periodic boundary conditions were applied in *x* and *y* directions, and a wall of helium atoms was placed on the top of the *z*-axis, which served as the fixed boundary condition.

After the construction of the initial model, geometry optimization was first adopted via Smart algorithms to eliminate high-energy configurations. An annealing procedure was performed with 4 cycles of NVT simulation with an initial temperature of 353 K and a mid-cycle temperature of 1000 K. Heating ramps per cycle and dynamics steps per ramp were set to 100 and 1000, respectively. After that, the wall was repositioned to ~10 nm position above the surface of carbon support, and a 5 ns NVT simulation at 353 K was performed to obtain the equilibrated ionomer configuration. Another 5 ns NVT simulation at 353 K was conducted to collect data for structural analysis.

To simplify the O_2_/N_2_ transport model and ensure sampling during MD simulations, we added 500 O_2_ molecules and 500 N_2_ molecules into the vacuum region above the ionomer surface to model the gas phase with a pressure of ~30 MPa [38,53], as shown in Figure 1d–g. Subsequently, a 5 ns NVT simulation at 353 K was performed to obtain the equilibrated state of the gas diffusion in the ionomer film. Finally, a 1 ns NVT simulation at 353 K was carried out to analyze the O_2_/N_2_ diffusion. It is worth noting that the current MD model focuses on molecular-level transport near TPB and is not intended to represent the complete electrochemical model of a PEMFC.

### 2.3. Analysis Method

#### 2.3.1. Radial Distribution Function

The radial distribution function (RDF), representing the probability of finding atom B from the reference atom A with the distance *r*, is defined as

(1) $$g_{A\text{-}B}\left(r\right)=\frac{\left\langle n_{B}\left(r\right)\right\rangle }{\rho_{B}\text{⋅}4\pi r^{2}dr}$$

where $\left\langle n_{B}\left(r\right)\right\rangle$ represents the average number of atoms B within a shell of thickness d*r* at a distance *r* from atom A; $\rho_{B}\;=\;N_{B}/V$ represents the density of atoms B in the system, $N_{B}$ is the total number of atoms B in the system and V is the total volume of the system.

#### 2.3.2. Solvent Surface Area to Volume Ratio

The solvent surface area to volume ratio of water clusters is defined as the ratio of its Connolly surface area to the occupied volume of water clusters, including water molecules and hydronium ions with a spherical probe of 0.14 nm. The formula is given as follows:

(2) $$\mathrm{SA}/\mathrm{OV}\;=\;\frac{\mathrm{Connolly}\;\mathrm{surface}\;\mathrm{area}}{\mathrm{Occupied}\;\mathrm{volume}}\;(r_{\mathrm{probe}}\;=\;0.14\;\mathrm{nm}),$$

#### 2.3.3. Diffusion Coefficient

The diffusion coefficient (*D*) is computed with the mean square displacement (MSD) as follows:

(3) $$\mathrm{MSD}(t)\;=\;\sum_{i=1}^{N}{\left|r_{i}(t)-r_{i}\left(0\right)\right|}^{2}/N,$$

(4) $$D=\underset{t\to \infty }{\lim}\mathrm{MSD}(t)/6t,$$

where $r_{i}(t)$ donates the position vectors of the *i*th particle at the moments *t*, and *N* represents the total number of a certain type of particles in the system.

## 3. Results and Discussion

### 3.1. Nanostructure of Hydrated Ionomers

**Figure 2:**
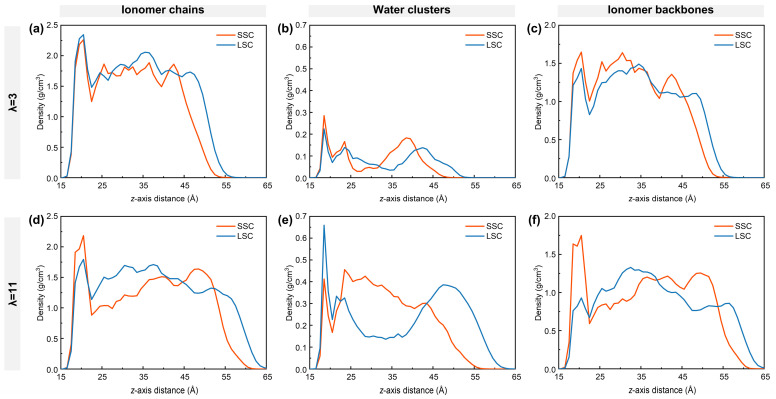
Density distribution along *z*-axis: (**a**,**d**) ionomer chains, (**b**,**e**) water clusters, and (**c**,**f**) ionomer backbones. (**a**–**c**) for λ = 3, (**d**–**f**) for λ = 11, respectively.

To understand the nanostructure of hydrated ionomers near TPBs, the density distribution of ionomer chains, water clusters and ionomer backbones of SSC and LSC ionomers under low- and high-hydration conditions are first examined as in Figure 2. The ionomer film formed on the carbon surface exhibits a thickness of approximately 4–5 nm, consisting of a dense layer adjacent to the graphite, a bulk region, and a surface region at 4 nm from the carbon surface [38,54,55]. At low hydration, the dense layer of the LSC PFSA shows a larger ionomer density (Figure 2a), mainly due to the direct adsorption of the ionomer chains onto the graphite. As the side-chain length increases from SSC to LSC, the spatial occupation of ionomers chains reduces the local water density in the dense layer (Figure 2b). In contrast, at high hydration, the ionomer density of the LSC ionomer in the dense layer is smaller than that of SSC ionomers, with the difference becoming more pronounced (Figure 2d). Comparison of Figure 2c,f reveals that adsorption of the LSC ionomers’ backbones on the graphite weakens significantly compared to SSC ionomers, making the LSC ionomer chains more flexible and prone to desorption. The resulting free volume near the graphite substrate in the LSC case is subsequently filled by water. Conversely, SSC ionomers and their backbones maintain relatively stable density in the dense layer under both hydration conditions, suggesting a more stable interfacial conformation.

**Figure 3:**
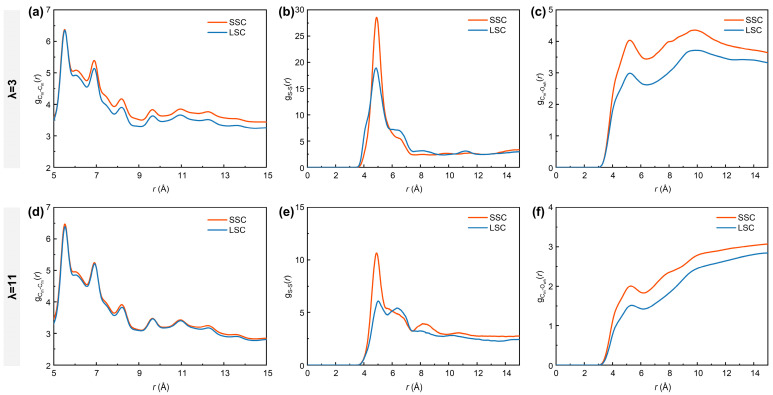
RDFs of (**a**,**d**) C_m_-C_m_ (carbon atoms in the ionomer backbones), (**b**,**e**) S-S and (**c**,**f**) C_m_-O_wh_ (oxygen atoms from water molecules and hydronium ions). (**a**–**c**) for λ = 3, (**d**–**f**) for λ = 11, respectively.

It is well-acknowledged that the bulk ionomers play a crucial role in mass transport [16,56]. To further characterize the nanostructure of the bulk ionomers, the RDFs of C_m_-C_m_ (carbon atoms in the ionomer backbones), S-S and C_m_-O_wh_ (oxygen atoms from the water molecules and hydronium ions) atom pairs under different hydration conditions in SSC and LSC systems are analyzed. As shown in Figure 3a,d, the curves of $g_{C_{m}\text{-}C_{m}}(r)$ exhibit overall higher intensities in SSC ionomers than in LSC ionomers. Similarly, as presented in Figure 3b,e, the $g_{S\text{-}S}(r)$ profiles display the most prominent peak at ~4.9 Å, with stronger intensities for SSC ionomers, indicating that their sulfonic acid groups tend to form more compact clusters. This behavior can be attributed to the reduced steric hindrance of shorter side-chains. Such reduced hindrance facilitates both intrachain folding and interchain packing. The intensities of $g_{C_{m}\text{-}C_{m}}(r)$ curves and $g_{S\text{-}S}(r)$ primary peaks are comparatively lower under higher hydration conditions due to the swollen hydrophilic regions and weakened interaction between sulfonic acid groups and water clusters [38]. Moreover, the $g_{C_{m}\text{-}O_{\mathrm{wh}}}(r)$ profiles (Figure 3c,f) reveal stronger curve intensities for SSC ionomers, reflecting an overall shortened distance between hydrophilic domains (sulfonic acid groups and water clusters) and the hydrophobic domains (PTFE backbones).

### 3.2. Transport Properties

**Figure 4:**
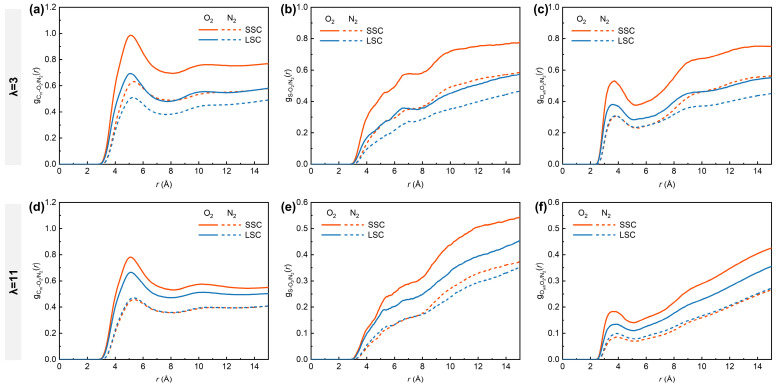
RDFs of (**a**,**d**) C_m_-O_2_/N_2_, (**b**,**e**) S-O_2_/N_2_, (**c**,**f**) O_wh_-O_2_/N_2_. (**a**–**c**) for λ = 3, (**d**–**f**) for λ = 11, respectively.

To understand the critical influence of the side-chains on gas distribution under low- and high-hydration conditions, we analyzed the RDFs between O_2_/N_2_ molecules and different parts of hydrated ionomers, represented by C_m_, S and O_wh_. As shown in Figure 4, the $g_{C_{m}\text{-}O_{2}}(r)$, $g_{S\text{-}O_{2}}(r)$ and $g_{O_{\mathrm{wh}}\text{-}O_{2}}(r)$ in SSC ionomers are consistently higher than those in LSC ionomers, with distinct peaks observed at ~5.1 Å for $g_{C_{m}\text{-}O_{2}}(r)$ (Figure 4a,d) and ~3.7 Å for $g_{O_{\mathrm{wh}}\text{-}O_{2}}(r)$ (Figure 4c,f). Previous MD studies have demonstrated that hydrophobic O_2_ molecules preferentially appear in free voids among hydrophobic backbones and interfacial regions between hydrophobic and hydrophilic domains [38,53], which provided insightful findings for analyzing how the structural differences in SSC and LSC ionomers influence gas transport. The more compact spatial aggregation of respective sulfonic groups and backbones in SSC ionomers tends to produce hydrophilic and hydrophobic phases of smaller scales, thereby enhancing the probability of direct contact between O_2_ and both domains. In addition, the closer packing of SSC ionomer backbones promotes the formation of denser hydrophobic regions that are favorable for O_2_ transport. With increasing hydration number from 3 to 11, however, the RDFs intensities between O_2_ and all reference atoms decrease considerably. This reduction originates from the swelling of highly hydrated ionomers, which creates more voids, thereby weakening the direct interaction between O_2_ and both hydrophilic and hydrophilic domains.

In contrast, the RDFs of N_2_ with all the reference atoms remain consistently lower than those of O_2_. Nevertheless, the hydration-dependent differences are evident for N_2_. Under low-hydration conditions, SSC ionomers exhibit higher $g_{C_{m}\text{-}N_{2}}(r)$ and $g_{S\text{-}N_{2}}(r)$ curve intensities compared to LSC ionomers, while $g_{O_{\mathrm{wh}}\text{-}N_{2}}(r)$ remains similar in the 2–7 Å range. This observation highlights the superior transport capability of the SSC ionomers’ hydrophobic frameworks for both gas molecules. Interestingly, SSC and LSC ionomers share nearly identical intensities for $g_{C_{m}\text{-}N_{2}}(r)$, $g_{S\text{-}N_{2}}(r)$ and $g_{O_{\mathrm{wh}}\text{-}N_{2}}(r)$ under high hydration conditions, while the disparity for O_2_ between SSC and LSC diminishes. Considering the lower solubility of N_2_ in water than O_2_ (solubility ratio in water at 101 325 Pa partial pressure of gas and 298.15 K: O_2_/N_2_ = 1.94) [57], the convergence of O_2_- and N_2_- related RDFs trends under high-hydration conditions can be interpreted as a consequence of water swelling, while the expansion of hydrophilic domains gradually partitions the hydrophobic matrix [10], thereby homogenizing gas transport pathways.

**Figure 5:**
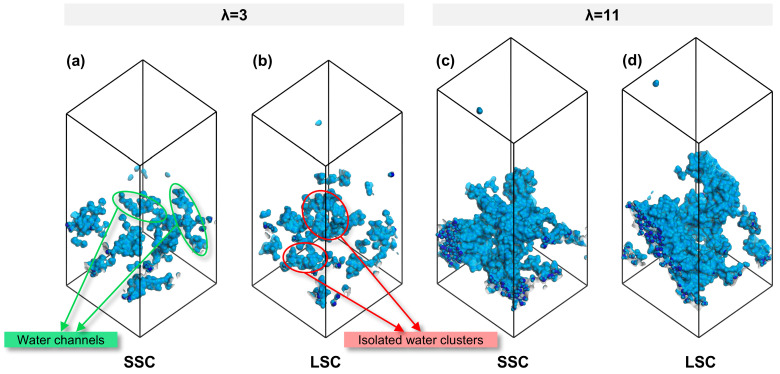
Water morphology in CLs of SSC and LSC PFSA, (**a**,**b**) λ = 3, (**c**,**d**) λ = 11.

To establish a connection between nanostructure and transport, we further analyzed the morphology of water clusters across different side-chains and hydration conditions. As summarized in Table 1, the SA/OV ratio of water clusters exhibit a smaller value at high hydration, consistent with previous study [38]. Notably, under the identical hydration conditions, water clusters in SSC ionomers consistently display lower SA/OV values than LSC ionomers, indicating a stronger tendency toward the formation of continuous water channels in SSC ionomers, whereas isolated water clusters are more likely to appear in LSC ionomers. The snapshots in Figure 5 visually confirm this trend. Such continuous water channels not only facilitate proton transport but also generate interconnected interfacial regions between hydrophilic and hydrophobic domains, which are favorable for gas migration.

**Figure 6:**
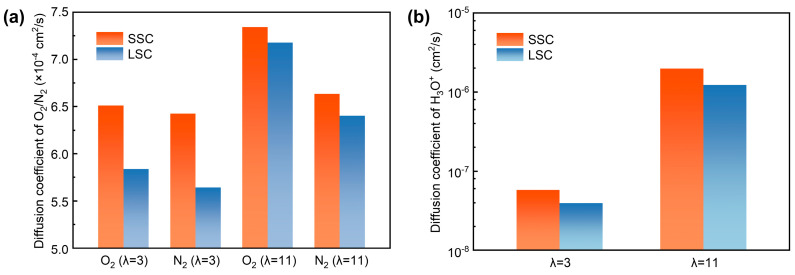
Diffusion coefficients, (**a**) O_2_/N_2_, (**b**) H_3_O^+^.

To quantitively analyze the transport, we computed the diffusion coefficients of O_2_, N_2_ and H_3_O^+^ in SSC and LSC ionomers under hydration conditions of 3 and 11. As presented in Figure 6a,b, both O_2_ and N_2_, as well as H_3_O^+^, exhibit higher diffusion coefficients under elevated hydration conditions, consistent with previous reports [38,58]. Due to the smaller kinetic diameter of O_2_ (O_2_ ~3.43 Å, N_2_ ~3.67 Å [59]) which indicates greater accessible free volume, it shows generally larger diffusion coefficients than N_2_. Importantly, the diffusion coefficients of O_2_ and N_2_ are systematically higher in SSC ionomers compared with LSC ionomers, which can be attributed to the continuous hydrophilic and hydrophobic interfacial regions which enhance gas transport. In addition, the differences between diffusion coefficients of gases in SSC and LSC systems are reduced at high hydration, indicating that swelling may lead to a relatively more homogenized gas transport pathway influenced by water as mentioned before. Nevertheless, SSC ionomers retain higher diffusion coefficients for H_3_O^+^ compared to LSC ionomers due to the persistent presence of continuous water channels.

## 4. Conclusions

In this study, MD simulations were conducted on CCL models comprising SSC and LSC ionomers in the coexistence of O_2_ and N_2_ to investigate the ionomer nanostructures and the transport properties of O_2_, N_2_ and H_3_O^+^ under different hydration conditions. The results indicate that side-chain structure exerts significant influences on the microscopic morphology of ionomer films and their associated transport behavior. Specifically, the RDFs of backbone carbon atoms and SA/OV suggest that SSC ionomers promote the formation of compact hydrophobic domains and well-connected water channels due to the reduced steric hindrance, giving rise to extended interfacial regions between hydrophilic and hydrophobic phases that serve as efficient pathways for O_2_ in comparison with LSC ionomers. At low hydration (λ = 3), structural features of SSC ionomers exhibit clear advantages over LSC ionomers, manifested in higher diffusion coefficients of O_2_, N_2_ and H_3_O^+^. However, swelling at high hydration (λ = 11) attenuates structural disparities between SSC and LSC ionomers, and water domains become a dominant factor governing transport. In addition, quantitative analysis confirms that O_2_ consistently possesses larger diffusion coefficients than N_2_, while both gases and H_3_O^+^ display higher diffusion coefficients under high-hydration conditions. Collectively, these findings provide molecular-level evidence that SSC enhances coupled oxygen and proton transport under practical cathode conditions, highlighting their potential in designing high-performance PEMFCs. Further studies with varying ionomer content and hydration conditions will be considered to bring a more comprehensive understanding related to side-chains.

## Figures and Tables

**Table 1:** SA/OV ratio of water clusters.

Ionomer Type	λ = 3	λ = 11
SSC ionomers	1.205	0.621
LSC ionomers	1.253	0.652

## Data Availability

Data is contained within the article.

## Abbreviations

The following abbreviations are used in this manuscript:

**Abbreviations**	
PEMFC	Proton Exchange Membrane Fuel Cell
MD	Molecular Dynamics
SSC	Short-Side-Chain
LSC	Long-Side-Chain
PFSA	Perfluorosulfonic Acid
PGM	Platinum-Group-Metal
ORR	Oxygen Reduction Reaction
CCL	Cathode Catalyst Layer
TPB	Triple-Phase Boundary
PTFE	Polytetrafluoroethylene
PPPM	Particle–Particle–Particle Mesh
vdW	van der Waals
RDF	Radial Distribution Function
SA/OV	Surface Area-to-Occupied Volume ratio
MSD	Mean Square Displacement
**Nomenclature**	
g(*r*)	Radial distribution function
$\left\langle n\right\rangle$	Average number of certain particles
ρ	Density of certain particles
*N*	Total number of certain particles in the system
*V*	Total volume of the system
*r*	Radial distance
d*r*	Shell thickness
$r_{\mathrm{probe}}$	Diameter of the spherical probe
*D*	Diffusion coefficient
λ	Hydration, which represents the ratio of the number of water molecules and hydronium ions to that of sulfonic groups
*t*	Simulation time
Å	Angstrom
